# Changes in tubular biomarkers with dietary intervention and metformin in patients with autosomal dominant polycystic kidney disease: a post-hoc analysis of two clinical trials

**DOI:** 10.1186/s12882-024-03643-6

**Published:** 2024-06-25

**Authors:** Wei Wang, Zhiying You, Cortney N. Steele, Berenice Gitomer, Michel Chonchol, Kristen L. Nowak

**Affiliations:** University of Colorado Anschutz Medical Campus, Aurora, CO USA

**Keywords:** ADPKD, Biomarkers, Clinical, Diet, Epidemiology, Metformin

## Abstract

**Background:**

Tubular biomarkers, which reflect tubular dysfunction or injury, are associated with incident chronic kidney disease and kidney function decline. Several tubular biomarkers have also been implicated in the progression of autosomal dominant polycystic kidney disease (ADPKD). We evaluated changes in multiple tubular biomarkers in four groups of patients with ADPKD who participated in one of two clinical trials (metformin therapy and diet-induced weight loss), based on evidence suggesting that such interventions could reduce tubule injury.

**Methods:**

66 participants (26 M/40 F) with ADPKD and an estimated glomerular filtration rate (eGFR) ≥ 30 ml/min/1.73m^2^ who participated in either a metformin clinical trial (*n* = 22 metformin; *n* = 23 placebo) or dietary weight loss study (*n* = 10 daily caloric restriction [DCR]; *n* = 11 intermittent fasting [IMF]) were included in assessments of urinary tubular biomarkers (kidney injury molecule-1 [KIM-1], fatty-acid binding protein [FABP], interleukin-18 [IL-18], monocyte chemoattractant protein-1 [MCP-1], neutrophil gelatinase-associated lipocalin [NGAL], clusterin, and human cartilage glycoprotein-40 [YKL-40]; normalized to urine creatinine), at baseline and 12 months. The association of baseline tubular biomarkers with both baseline and change in height-adjusted total kidney volume (HtTKV; percent change from baseline to 12 months) and estimated glomerular filtration rate (eGFR; absolute change at 12 months vs. baseline), with covariate adjustment, was also assessed using multiple linear regression.

**Results:**

Mean *±* s.d. age was 48 ± 8 years, eGFR was 71 ± 16 ml/min/1.73m^2^, and baseline BMI was 30.5 ± 5.9 kg/m^2^. None of the tubular biomarkers changed with any intervention as compared to placebo. Additionally, baseline tubular biomarkers were not associated with either baseline or change in eGFR or HtTKV over 12 months, after adjustments for demographics, group assignment, and clinical characteristics.

**Conclusions:**

Tubular biomarkers did not change with dietary-induced weight loss or metformin, nor did they associate with kidney disease progression, in this cohort of patients with ADPKD.

**Supplementary Information:**

The online version contains supplementary material available at 10.1186/s12882-024-03643-6.

## Background

Autosomal dominant polycystic kidney disease (ADPKD) is characterized by continuous enlargement of cysts and loss of kidney function, leading to end stage kidney diseases requiring dialysis or transplant in the majority of patients [1]. Disease severity is variable in affected individuals even within members of the same family [2]. Prognostic markers are important for monitoring disease progression and efficacy of novel therapy development, especially in the early stages of ADPKD where kidney function remains relatively unaffected [3, 4]. Currently, height adjusted TKV (HtTKV) is the gold standard for that purpose [5], but it is expensive and time consuming. There has been considerable interest in developing more convenient biomarkers for ADPKD kidney disease. Tubular biomarkers reflecting tubular injury and repair are associated with incident chronic kidney disease (CKD) and kidney function decline [6]. Cyst formation in ADPKD involves abnormal tubular cell proliferation and fluid secretion resulting in diminished tubular reabsorption, upregulation of tubular proteins, and release of cytokines from inflammatory cells [7–9]. Circulatory and urinary biomarkers have been related to kidney growth and/or function in patients with ADPKD; however, results are inconsistent [3, 4, 10–12].

Accumulating evidence indicates that obesity is an independent risk factor for the development and progression of kidney diseases [13], including ADPKD [14, 15]. We were the first to show that overweight, and particularly obesity are strong independent predictors for ADPKD disease progression in humans [14, 15]. We also demonstrated that daily caloric restriction (DCR) and intermittent fasting (IMF) resulted in significant weight loss, which strongly correlated with slower kidney growth [16]. Furthermore, weight loss (through diet or bariatric surgery) has been shown to change urinary or circulating tubular biomarkers in adults with obesity who do not have kidney disease [17–19].

Metformin, an FDA approved medication for treatment of type 2 diabetes, improves kidney outcomes in PKD mouse models [20, 21]. Metformin also improves kidney injury markers in various disease mouse models, including PKD [20, 22–24], with a few human biomarker studies in non-PKD patients [25, 26]. Our recently completed clinical trial of metformin in ADPKD patients was designed to evaluate its safety and feasibility [27]. Interestingly, metformin slowed kidney growth significantly as compared to the placebo group in a subgroup of patients with baseline HtTKV greater than 800 ml/m.

We therefore hypothesized that in patients with ADPKD, urinary tubular biomarkers independently associate with HtTKV and/or eGFR, and interventions through either diet (DCR or IMF) or metformin reduce tubular injury and thus urinary tubular biomarkers. Urine samples from the two aforementioned studies [16, 27] were examined to test our hypothesis. The goals of this study were to (1) determine the association of urinary tubular biomarkers with HtTKV and eGFR (at baseline and change over 12 months); (2) determine changes in urinary tubular biomarkers in response to DCR, IMF, metformin, and placebo.

## Methods

### Study design

The details of each of the clinical trials have been reported previously [16, 27] and are described in brief. Both trials took place at the University of Colorado Anschutz Medical Campus and included national recruitment of study participants. The assessment of predictor and outcome variables was identical in both studies, and inclusion/exclusion criteria and participant characteristics were very similar. Both trials were approved by the Institutional Review Board of the University of Colorado Anschutz Medical Campus and adhere to the *Declaration of Helsinki*. The nature, benefits and risks of the study were explained to the volunteers and their written informed consent /assent was obtained prior to participation.

#### DCR and IMF

This 12 month, pilot, randomized behavioral weight loss intervention study included DCR (34% daily restriction) or IMF (fasting goal of 20% of caloric needs for weight maintenance three non-consecutive days per week) in non-diabetic adults 18–65 years of age with overweight or obesity and ADPKD with normal to moderately declined kidney function (eGFR ≥ 30 ml/min/1.73m^2^) (NCT03342742) [16]. The trial aimed to evaluate the safety, acceptability, and tolerability of each intervention. Changes in HtTKV and eGFR and their association with the change in weight were exploratory outcomes. Participants were enrolled between September 2018 through August 2019. Twenty-eight participants were randomized to either DCR or IMF, and 10 DCR and 11 IMF participants completed the study with stored urine samples available, and thus, were included in the current analysis.

#### Metformin

This was a 12 month, pilot, randomized, placebo controlled trial designed to examine the safety and tolerability of metformin in ADPKD patients aged 30–60 years without diabetes and with an estimated glomerular filtration (eGFR) 50-80 ml/min/1.73m^2^ (NCT02903511) [27]. Changes in HtTKV and eGFR and their association were assessed as exploratory outcomes. Participants were enrolled between November 2016 and September 2019, with 51 participants randomized. The starting dose of metformin was 500 mg twice a day, and the dose was up-titrated every two weeks by 500 mg to a maximal dose of 1,000 mg twice a day if well-tolerated. Twenty-two and twenty-three participants in the metformin and control group, respectively, completed the trial and were included in the current study.

### Sample collection

Spot urine samples were collected at baseline and 12 months after intervention in both studies. All samples were collected in the morning following an overnight fast. Urine was aliquoted, frozen immediately, and stored at -80 °C until the day of analyses.

### Measurement of urinary biomarkers

Urinary biomarkers were measured using stored spot urine samples collected at baseline and 12 months. Specifically, markers measured were: kidney injury molecule-1 [KIM-1], fatty-acid binding protein [FABP], interleukin-18 [IL-18], monocyte chemoattractant protein-1 [MCP-1], neutrophil gelatinase-associated lipocalin [NGAL], clusterin, and human cartilage glycoprotein-40 [YKL-40]. Customized multiplex U-plex ELISA kits from Meso Scale Discovery (Rockville, MD) were used and assays were performed in duplicates. Urine was diluted 1:2 for IL-18, MCP-1, KIM-1, clusterin, FABP4 assay and 1:50 for NGAL and YKL-40 assays. All values were normalized to urine creatinine. Urine creatinine was measured in the clinical lab at the University Colorado Hospital.

### Measurement of total kidney volume

A Siemens Skyra 3.0T system was used to obtain an abdominal MRI at baseline and 12 months, as described in the original studies [16, 27]. TKV was measured using Analyze software (Analyze 11.0, Mayo Foundation, Rochester, MN) by a single blinded investigator for each study. Annual percent change in HtTKV was calculated based on actual number of months between the two time points. Disease severity was categorized according to the Mayo Imaging Classification system [28].

### Estimated glomerular filtration rate

Estimated glomerular filtration rate was measured using the CKD-EPI equation [29], based on serum creatinine determinations using a Beckman Coulter AU5800 in the central laboratory of the University of Colorado Hospital. Absolute change in eGFR at 12 months as compared to baseline was calculated.

### Assessment of covariates

Race was collected in accordance to NIH guidelines, self-reported, and collapsed into White and non-White categories given the low enrollment of non-White participants. Blood pressure was measured in triplicate in the seated position after five minutes of quiet rest (Omron HEM 907XL). Body weight was measured on a calibrated digital scale to the nearest 0.1 kg and height was measured to the nearest 1 mm using a stadiometer to calculate body mass index. Serum glucose was measured in a fasted state by the University Colorado Hospital clinical lab.

### Statistical analyses

Baseline characteristics are presented as means ± s.d. or median (interquartile range) for continuous variables and *n* (%) for categorical variables. Skewed variables were log-transformed in all analyses (HtTKV and all tubular biomarkers). ANOVA with Tukey’s Studentized Range [HSD] post-hoc test was used to compare differences in baseline characteristics and tubular biomarkers across the four groups. Univariate associations between tubular biomarkers and baseline and change in HtTKV/eGFR were plotted graphically with Pearson’s bivariate correlations. The association (β [95% Confidence Interval]) of urinary tubular biomarkers with (1) baseline HtTKV, and (2) baseline eGFR was assessed using multiple linear regression models adjusted for treatment group (model 1), plus age and sex (model 2), plus systolic blood pressure and body-mass index (BMI) (model 3), plus baseline eGFR (for the HtTKV outcome) or baseline HtTKV (for the eGFR outcome) (model 4). Identical models were used to determine the association of each tubular biomarker with (1) percentage change in HtTKV at 12 months, and (2) absolute change in eGFR at 12 months (both baseline eGFR and baseline HtTKV were included in model 4). All statistical analyses were performed using SAS version 9.4 (SAS Institute, Cary, NC).

## Results

### Study participants and baseline clinical characteristics

Participant characteristics are shown in Table 1. Mean age was 48 ± 8 years, eGFR was 70 ± 16 ml/min/1.73m^2^, and baseline BMI was 30.6 ± 6.0 kg/m^2^. A total of 66 participants (26 M/40 F) with ADPKD were included, with 45 in the metformin clinical trial (*n* = 22 metformin; *n* = 23 placebo control) and 21 in the dietary weight loss study (*n* = 10 in DCR; *n* = 11 in IMF). There were no significant differences among the four groups regarding baseline eGFR, blood pressure, serum glucose levels, HtTKV, and Mayo class classification [30]. BMI was significantly higher in the IMF group vs. metformin or control, due to the inclusion criteria for the weight loss study.

**Table 1:** Demographics and Clinical Characteristics

Variable	All (*n* = 66)	Control (*n* = 23)	Metformin (*n* = 22)	DCR (*n* = 10)	IMF (*n* = 11)
**Age**, y	48 ± 8	49 ± 7	48 ± 7	47 ± 13	47 ± 6
**Sex**, *n* (%) Male	26 (39%)	8 (36%)	18 (36%)	5 (50%)	5 (45%)
**Race**, *n* (%) White	61 (92%)	21 (91%)	21 (95%)	10 (100%)	9 (82%)
**CKD-EPI eGFR**, mL/min/1.73m^2^	70 ± 16	73 ± 13	69 ± 14	63 ± 22	75 ± 17
**Systolic BP**, mmHg	123 ± 13	124 ± 12	124 ± 12	114 ± 13	126 ± 12
**Diastolic BP**, mmHg	80 ± 8	81 ± 8	81 ± 7	74 ± 9	83 ± 4
**BMI**, kg/m^2^	30.6 ± 6.0	28.3 ± 4.3	29.7 ± 7.0	34.1 ± 5.8	34.2 ± 4.7 *
**Serum glucose**, mg/dL	94 ± 9	93 ± 8	94 ± 9	93 ± 10	94 ± 9
**HtTKV**, mL/m	742 (533, 1,349)	647 (500, 1,052)	1,125 (532, 1,124)	840 (472, 1,122)	1,221 (394, 2,204)
**Mayo Class**					
Class A	5 (8%)	2 (9%)	1 (5%)	0 (0%)	2 (18%)
Class B	16 (25%)	7 (30%)	6 (27%)	2 (22%)	1 (9%)
Class C	30 (46%)	13 (57%)	7 (32%)	5 (56%)	5 (45%)
Class D	8 (12%)	1 (5%)	5 (23%)	1 (11%)	1 (9%)
Class E	6 (9%)	0 (0%)	3 (14%)	1 (11%)	2 (18%)
**Annual Δ eGFR**, ml/min/1.73m^2^ per yr	-2.0 (-7.0, 4.0)	-2.5 (-8.0, 2.0)	1.0 (-7.0, 6.0)	1.0 (-7.0, 4.0)	-5.0 (-8.0, 13.0)
**Annual Δ lnHtTKV**, %	3.5 (-1.0, 6.5)	5.2 (-0.2, 6.3)	3.5 (1.3, 6.5)	1.5 (-0.3, 2.8)	0.0 (-3.3, 11.4)

* *p* < 0.05 vs. control or metformin (ANOVA with Tukey’s Studentized Range [HSD] post-hoc test). Log-transformed values were used in statistical comparisons for HtTKV. *N* = 1 DCR missing MRI imaging

### Association of baseline tubular biomarkers with baseline and change in height-adjusted TKV and eGFR

In cross-sectional analyses including all participants across both studies, several baseline tubular biomarkers (log-adjusted) were associated with baseline HtTKV (log-adjusted) or eGFR (Supplemental Fig. 1), however, no associations remained significant after adjustment for covariates (Table 2). Only MCP-1 was associated with change in eGFR after 12 months of diet or metformin intervention (Supplemental Fig. 2); however, again, no tubular biomarkers were significantly associated with change in either HtTKV or eGFR at 12 months after adjustments for demographics, group assignment, and clinical characteristics (Table 3).

**Table 2:** Association (β [95% confidence interval]) of baseline tubular biomarkers (log-adjusted) with baseline height-adjusted total kidney volume (log-adjusted) and estimated glomerular filtration rate in all participants combined

Variable	HtTKV				eGFR			
	Model 1	Model 2	Model 3	Model 4	Model 1	Model 2	Model 3	Model 4
**KIM-1**	0.20 (-0.25, 0.65)	0.16 (-0.28, 0.61)	0.13 (-0.33, 0.58)	0.09 (-0.36, 0.55)	-6.7 (-17.7, 4.3)	-4.7 (-15.1, 5.7)	-3.6 (-13.9, 6.6)	-3.1 (-13.3, 7.1)
**FABP**	0.06 (-0.24, 0.35)	0.07 (-0.22, 0.36)	0.03 (-0.28, 0.34)	-0.01 (-0.32, 0.30)	-3.5 (-10.1, 3.0)	-3.8 (-9.9, 2.3)	-3.9 (-10.2, 2.4)	-3.7 (-9.9, 2.5)
**IL-18**	-0.24 (-0.75, 0.27)	-0.28 (-0.80, 0.24)	-0.32 (-0.86, 0.21)	-0.31 (-0.84, 0.22)	-2.4 (-14.7, 9.9)	0.41 (-11.3, 12.1)	1.9 (-10.2, 13.9)	0.60 (-11.5, 12.7)
**MCP-1**	0.14 (-0.20, 0.47)	0.13 (-0.20, 0.46)	0.18 (-0.17, 0.53)	0.17 (-0.18, 0.52)	-2.6 (-10.9, 5.6)	-1.7 (-9.3, 5.9)	-1.5 (-9.4, 6.4)	-1.2 (-9.2, 6.9)
**NGAL**	-0.05 (-0.28, 0.20)	-0.05 (-0.31, 0.21)	-0.07 (-0.34, 0.20)	-0.06 (-0.33, 0.21)	-1.0 (-6.5, 4.5)	0.05 (-5.5, 5.6)	0.74 (-4.9, 6.4)	0.46 (-5.2, 6.1)
**Clusterin**	-0.05 (-0.41, 0.32)	-0.01 (-0.38, 0.36)	-0.01 (-0.38, 0.36)	-0.07 (-0.45, 0.30)	-4.2 (-12.8, 4.4)	-4.4 (-12.5, 3.7)	-3.4 (-11.6, 4.8)	-3.6 (-11.7, 4.5)
**YKL-40**	-0.20 (-0.42, 0.02)	-0.22 (-0.44, 0.00)	-0.21 (-0.43, 0.01)	-0.19 (-0.41, 0.04)	1.7 (-3.4, 6.8)	2.7 (-2.1, 7.6)	2.4 (-2.4, 7.3)	1.6 (-3.4, 6.6)

Model 1 is adjusted for treatment group

Model 2 is adjusted for model 1 + age, sex

Model 3 is adjusted for model 2 + systolic blood pressure, body mass-index

Model 4 is adjusted for model 3 + baseline eGFR or baseline height-adjusted total kidney volume (log-adjusted)

HtTKV, height-adjusted total kidney volume; eGFR, estimated glomerular filtration rate; KIM-1, kidney injury molecule-1; FABP, fatty-acid binding protein; IL-18, interleukin-18; MCP-1, monocyte chemoattractant protein-1; NGAL, neutrophil gelatinase-associated lipocalin; YKL-40, human cartilage glycoprotein-40

**Table 3:** Association (β [95% confidence interval]) of baseline tubular biomarkers (log-adjusted) with percent change in height-adjusted total kidney volume (log-adjusted) and absolute change in estimated glomerular filtration rate at 12 months in all participants combined

Variable	HtTKV				eGFR			
	Model 1	Model 2	Model 3	Model 4	Model 1	Model 2	Model 3	Model 4
**KIM-1**	4.1 (-0.13, 13.8)	4.0 (-1.3, 9.3)	1.9 (-4.7, 8.4)	2.0 (-4.7, 8.6)	0.42 (-5.8, 6.6)	0.36 (-5.9, 6.7)	1.5 (-4.9, 7.9)	2.3 (-4.0, 8.6)
**FABP**	0.68 (-2.7, 4.1)	0.45 (-3.0, 3.9)	-1.8 (-6.1, 2.5)	-1.5 (-6.0, 2.9)	-1.4 (-3.1, 0.41)	-2.0 (-5.8, 1.8)	-1.2 (-5.3, 2.9)	-0.71 (-4.8, 3.4)
**IL-18**	1.4 (-5.9, 8.6)	0.14 (-7.4, 7.6)	-4.9 (-12.2, 2.4)	-6.0 (-13.5, 1.4)	-6.5 (-13.1, 0.06)	-6.9 (-13.6, -0.22)	-5.5 (-12.6, 1.7)	-6.6 (-13.7, 0.43)
**MCP-1**	2.8 (-4.2, 9.8)	2.5 (-4.5, 9.5)	-0.34 (-5.8, 5.1)	-0.39 (-6.0, 5.2)	-2.5 (-7.4, 2.3)	-2.4 (-7.3, 2.5)	-1.9 (-7.1, 3.4)	-1.3 (-6.7, 4.0)
**NGAL**	1.4 (-2.1, 4.9)	0.92 (-2.6, 4.4)	0.62 (-3.1, 4.3)	0.46 (-3.3, 4.2)	-1.4 (-4.6, 1.8)	-1.5 (-5.0, 2.0)	-0.50 (-4.2, 3.2)	-0.78 (-4.4, 2.8)
**Clusterin**	3.0 (-3.5, 9.4)	2.5 (-4.1, 19.1)	2.4 (-2.7, 7.6)	2.7 (-2.6, 8.0)	0.23 (-4.4, 4.8)	0.95 (-3.8, 5.7)	1.9 (-3.0, 6.8)	2.1 (-2.8, 7.0)
**YKL-40**	-0.37 (-3.2, 2.5)	-0.35 (-3.2, 2.5)	-0.28 (-3.6, 3.0)	-0.44 (-3.9, 3.06)	-0.86 (-3.9, 2.1)	-0.83 (-3.9, 2.2)	-0.54 (-3.7, 2.6)	-1.5 (-4.7, 1.8)

Model 1 is adjusted for treatment group

Model 2 is adjusted for model 1 + age, sex

Model 3 is adjusted for model 2 + systolic blood pressure, body-mass index

Model 4 is adjusted for model 3 + baseline eGFR, baseline height-adjusted total kidney volume (log-adjusted)

HtTKV, height-adjusted total kidney volume; eGFR, estimated glomerular filtration rate; KIM-1, kidney injury molecule-1; FABP, fatty-acid binding protein; IL-18, interleukin-18; MCP-1, monocyte chemoattractant protein-1; NGAL, neutrophil gelatinase-associated lipocalin; YKL-40, human cartilage glycoprotein-40

N=1 participant is missing change in eGFR and N=4 are missing change in HtTKV

### Changes in urinary levels of tubular biomarkers with metformin, DCR, IMF and placebo

**Fig. 1:**
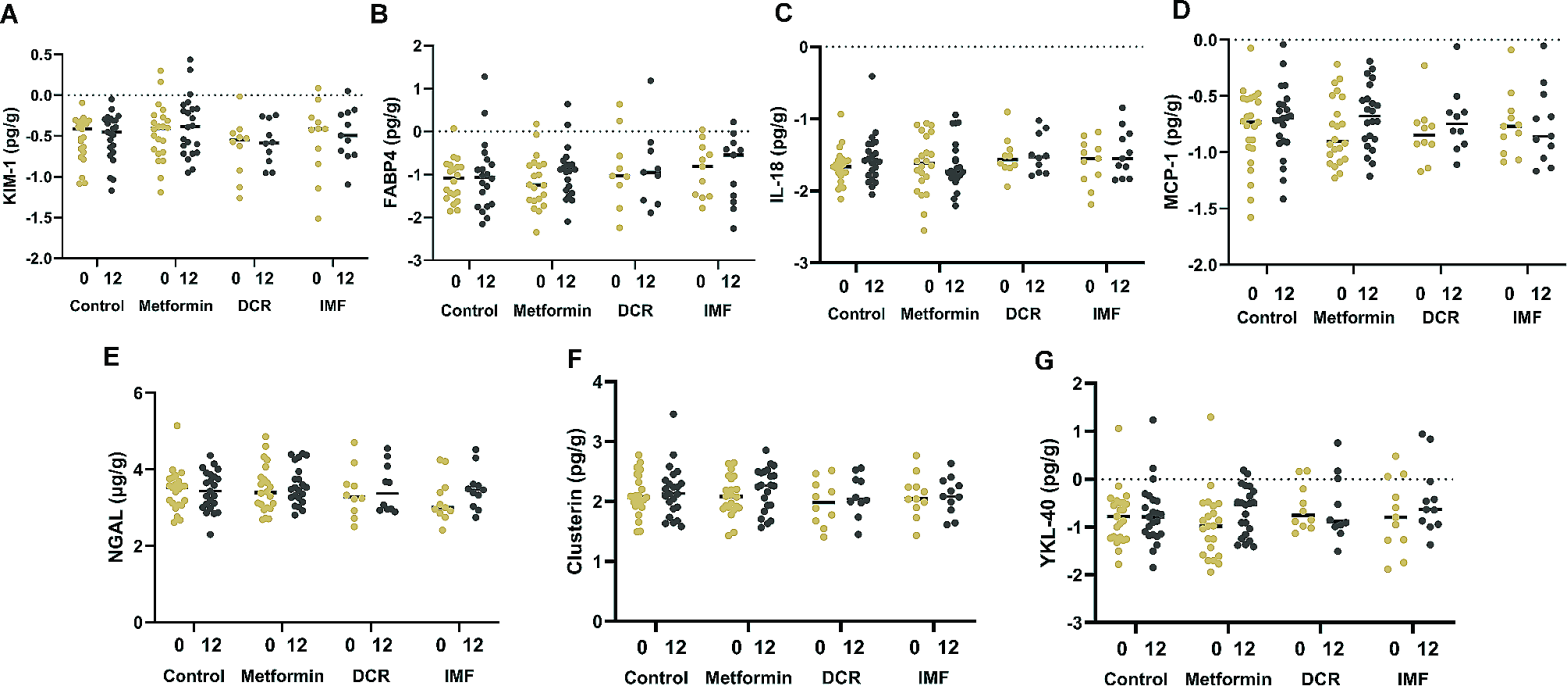
Urinary concentrations of kidney injury molecule-1 (KIM-1; **Panel A**); fatty-acid binding protein 4 (FABP4; **Panel B**); interleukin-18 (IL-18; **Panel C**); monocyte chemoattractant protein-1 (MCP-1; **Panel D**); neutrophil gelatinase-associated lipocalin (NGAL; **Panel E**); clusterin (**Panel F**); human cartilage glycoprotein-40 (YKL-40; **Panel G**), normalized to urine creatinine and log-transformed, at baseline (month 0, gold) and 12 months (dark gray). Groups are control, metformin, daily caloric restriction (DCR), and intermittent fasting (IMF). Horizontal lines represent median and individual data points are shown as circles

Baseline and month 12 urinary KIM-1, FABP4, IL-18, MCP-1, NGAL, Clusterin, and YKL-40, all normalized to urinary creatinine, are displayed graphically as individual data points and median values for each of the four groups in Fig. 1. The specific values (median [interquartile range]) from this figure are presented in Supplemental Table 1. None of the tubular biomarkers were changed significantly after 12-months of intervention with either weight loss (DCR or IMF) or metformin as compared to placebo control.

## Discussion

We conducted urinary assessments of multiple tubular biomarkers including KIM-1, NGAL, IL-18, MCP-1, clusterin, FABP4, and YKL-40 in ADPKD patients with moderately preserved eGFR who participated in weight loss (IMF or DCR) or metformin safety and feasibility studies. Contrary to our hypothesis, we observed no association of any tubular biomarker with baseline kidney disease severity or longitudinal measures of kidney disease progression (eGFR and HtTKV), nor any changes in tubular biomarkers with dietary or metformin intervention, after adjustment for covariates. This is despite the fact that DCR and IMF appeared to reduce HtTKV as compared to historical controls [16], and metformin slowed kidney growth in a sub-group of participants with HtTKV ≥ 800 ml/m) in the parent studies [27].

We used a combination of multiple biomarkers in the current study to capture multiple dimensions of kidney tubular health. Among the biomarkers, KIM-1, NGAL, IL-18, FABP-4 and clusterin are considered kidney tubular injury biomarkers, and MCP-1 and YKL-40 are related to inflammation and fibrosis. They have been extensively studied in acute and chronic kidney diseases [31–34], with additional studies in ADPKD patients [3, 11, 35–37]. Notably, changes in urinary tubular biomarkers in response to dietary or metformin intervention have not been reported previously in humans with ADPKD, although studies in murine models of PKD [20, 38] and in humans without ADPKD [39] support our hypothesis that changes may occur in response to weight loss and/or metformin.

The circulating inflammatory biomarker IL-18 is produced by human adipose tissue [40]. We previously reported a significant reduction in serum IL-18 levels with DCR at 12 months [16]. However, in the current study, urinary IL-18 was not changed with weight loss. The circulation may not reflect excretion by the kidney. The lack of association of urine IL-18 with kidney disease severity is in agreement with the results from the CRISP study, where urinary IL-18 did not associate with changes in eGFR or HtTKV in ADPKD patients with preserved kidney function [10].

Importantly, physiological differences may be present between urine and the circulation. Disagreement between urinary and circulating levels of KIM-1 were also observed in a case-control analysis from the ACCORD trial (the Action to Control Cardiovascular Disease Trial), conducted in patients with type 2 diabetes. In this study, plasma [41] but not urinary KIM-1 was associated with eGFR reduction [34]. The discrepancy between the circulating and urine levels of biomarkers might be an indication of lack of communication between the cysts and the urinary collecting system. The lack of association in the current study might be also due to its small sample size limiting the power. Indeed, several previous studies have shown an association between urinary biomarkers and ADPKD kidney disease progression [3, 11, 35–37]. Different patient populations, the extent of kidney dysfunction, relatively short study duration, and the nature of biomarkers may have contributed to our discrepant results. Whether circulating levels of biomarkers are better indicators of kidney disease progression than urine in ADPKD requires further investigation.

The biomarkers in the current study were expressed as their ratios to urine creatinine. The reason for the adjustment is to control for urine flow rate. However, urine creatinine not only assesses urine toxicity, but also indicates differences in muscle mass [42], and its secretion is influenced by eGFR [43]. 1/Ucr itself is an index of adverse clinical outcomes in ambulatory individuals [44]. Interestingly, the association between urinary NGAL and incident CKD stage 3 was attenuated after adjustment for urinary creatinine concentration in a population-based cohort of middle-aged and older adults [45]. In contrast, in another cohort of middle-aged and older adults free from baseline cardiovascular disease, urinary KIM-1 was associated with incident CKD stage 3 with or without standardizing to urinary creatinine [46]. Timed excretion of urinary biomarkers has been suggested by Waikar et al. [43], but could be challenging to implement. Another alternative is to use urine creatinine as a separate covariate in models [47]. In the current study, a spot urine sample was collected, aliquoted, frozen immediately and stored in a -80 degree freezer until analyses. Thus, the integrity of the biomarkers was likely intact.

The major strength of our study is that changes in tubular biomarkers in ADPKD patients in response to an intervention have not been measured previously, with the exception of a small cohort treated with tolvaptan [48]. Our study cohort included patients with a wide spectrum of disease severity ranging from Mayo class A to E, with the majority (70%) having moderate disease (class B and C), and 21% in the most advanced groups (class D and E). This is fairly representative of the general ADPKD population, but the limited sample with the most severe categorization may have limited the ability to detect associations. Additional strengths include measuring a combination of urinary biomarkers as opposed to a single biomarker (including several that have not been evaluated previously in relation to kidney disease progression in patients with ADPKD), providing a more thorough assessment of tubular health. Additionally, the setting of clinical trials allowed for well controlled assessment of outcome variables and covariates. Limitations of the study include a relatively small sample size limiting statistical power, relatively short study duration, and the absence of a normal control group as a comparator. An earlier time point prior to month 12 may have allowed us to detect changes in tubular biomarkers, as the effect of tolvaptan was most apparent during the early period of the intervention in a prior small study [48]; however, we did not have urine samples available for analysis at an earlier time point. Additionally, as the measured urinary biomarkers represent renal damage that is most often reflected by a loss in eGFR, the lack of benefit of the therapies in the parent trials on eGFR may have limited the ability to detect changes in urinary tubular biomarkers.

## Conclusions

In summary, tubular biomarkers did not change with dietary-induced weight loss or metformin, nor were they associated with kidney disease severity or progression, in this cohort of patients with ADPKD, many of whom had overweight or obesity. A larger cohort is needed in the future to more thoroughly assess changes in kidney tubular health in response to lifestyle or pharmacological intervention in patients with ADPKD.

### Electronic supplementary material

Below is the link to the electronic supplementary material.

Supplementary Material 1

Supplementary Material 2

Supplementary Material 3

## Data Availability

The data from the dietary studies are available at Zenodo: 10.5281/zenodo.11152188.
