# Supplementary material for: Changes in tubular biomarkers with dietary intervention and metformin in patients with autosomal dominant polycystic kidney disease: a post-hoc analysis of two clinical trials
Source: BMC Nephrol. 2024 Jun 25;25:206. doi: 10.1186/s12882-024-03643-6 (PMC11200847; doi:10.1186/s12882-024-03643-6)
Supplement: Supplementary file 1 — Supplementary Material 1 [file 12882_2024_3643_MOESM1_ESM.docx]

**Supplemental Table 1:** Change Tubular Biomarkers with Metformin, Daily Caloric Restriction, Intermittent Fasting, and Placebo Control

| **Variable** | **Control**  (n=23) | | **Metformin**  (n=22) | | **DCR**  (n=10) | | **IMF**  (n=11) | | |  |
| --- | --- | --- | --- | --- | --- | --- | --- | --- | --- | --- |
|  | **Baseline** | **Month 12** | **Baseline** | **Month 12** | **Baseline** | **Month 12** | **Baseline** | **Month 12** | |  |
| **UACR** |  |  |  |  | 14.79 (9.84, 22.48) | 17.78 (11.91, 34.03) | 11.22 (7.09, 13.46) | | 17.07 (10.00, 33.33) | |
| **UPCR** | 0.014 (0.010, 0.037) | 0.019 (0.012, 0.031) | 0.017 (0.010, 0.038) | 0.016 (0.009, 0.042) |  |  |  |  | |  |
| **KIM-1** (pg/g) | 0.38 (0.20, 0.48) | 0.35 (0.20, 0.50) | 0.39 (0.20, 0.53) | 0.41 (0.20, 0.68) | 0.28 (0.12, 0.34) | 0.23 (0.11, 0.32) | 0.38 (0.14, 0.53) | 0.32 (0.19, 0.61) | |  |
| **FABP** (pg/g) | 0.04 (0.03, 0.13) | 0.07 (0.02, 0.17) | 0.06 (0.03, 0.16) | 0.13 (0.04, 0.17) | 0.09 (0.02, 0.27) | 0.10 (0.02, 0.35) | 0.22 (0.03, 0.42) | 0.28 (0.02, 0.54) | |  |
| **IL-18** (pg/g) | 0.02 (0.02. 0.03) | 0.03 (0.02. 0.04) | 0.02 (0.01. 0.06) | 0.02 (0.02. 0.04) | 0.03 (0.02. 0.04) | 0.03 (0.02. 0.06) | 0.03 (0.01. 0.04) | 0.02 (0.01. 0.06) | |  |
| **MCP-1** (pg/g) | 0.18 (0.11, 0.30) | 0.20 (0.12, 0.27) | 0.12 (0.09, 0.31) | 0.21 (0.13, 0.30) | 0.14 (0.12, 0.18) | 0.18 (0.12, 0.23) | 0.17 (0.10, 0.23) | 0.14 (0.24, 0.09) | |  |
| **NGAL** (μg/g) | 3361 (1187, 5689) | 2697 (1020, 7447) | 2506 (993, 6269) | 3203 (1808, 8870) | 1935 (836, 4079) | 11,448 (859, 12,115) | 1008 (711, 3370) | 2925 (1644, 4158) | |  |
| **Clusterin** (pg/g) | 116.9 (83.5, 270.5) | 135.5 (75.8, 226.3) | 120.5 (78.7, 240.3) | 185.1 (85.9, 312.7) | 99.0 (47.5, 189.3) | 104.0 (95.1, 230.5) | 109.6 (79.5, 175.0) | 121.1 (72.0, 223.2) | |  |
| **YKL-40** (pg/g) | 0.17 (0.06, 0.32) | 0.16 (0.07, 0.37) | 0.11 (0.03, 0.27) | 0.30 (0.07, 0.55) | 0.18 (0.10, 0.63) | 0.14 (0.11, 1.03) | 0.16 (0.05, 1.14) | 0.23 (0.11, 0.90) | |  |

All urinary tubular biomarkers are normalized to urine creatinine. DCR, daily caloric restriction; IMF, intermittent fasting. UACR, urinary albumin-to-creatinine ratio, PCR, urinary protein-to-creatinine ratio, KIM-1, kidney injury molecule-1; FABP, fatty-acid binding protein; IL-18, interleukin-18; MCP-1, monocyte chemoattractant protein-1; NGAL, neutrophil gelatinase-associated lipocalin; YKL-40, human cartilage glycoprotein-40.
