# Supplementary material for: Changes in tubular biomarkers with dietary intervention and metformin in patients with autosomal dominant polycystic kidney disease: a post-hoc analysis of two clinical trials
Source: BMC Nephrol. 2024 Jun 25;25:206. doi: 10.1186/s12882-024-03643-6 (PMC11200847; doi:10.1186/s12882-024-03643-6)
Supplement: Supplementary file 2 — Supplementary Material 2 [file 12882_2024_3643_MOESM2_ESM.docx]

**Supplemental Figure 1.** Associations between urinary tubular biomarkers and height-adjusted total kidney volume and estimated glomerular filtration rate at baseline.
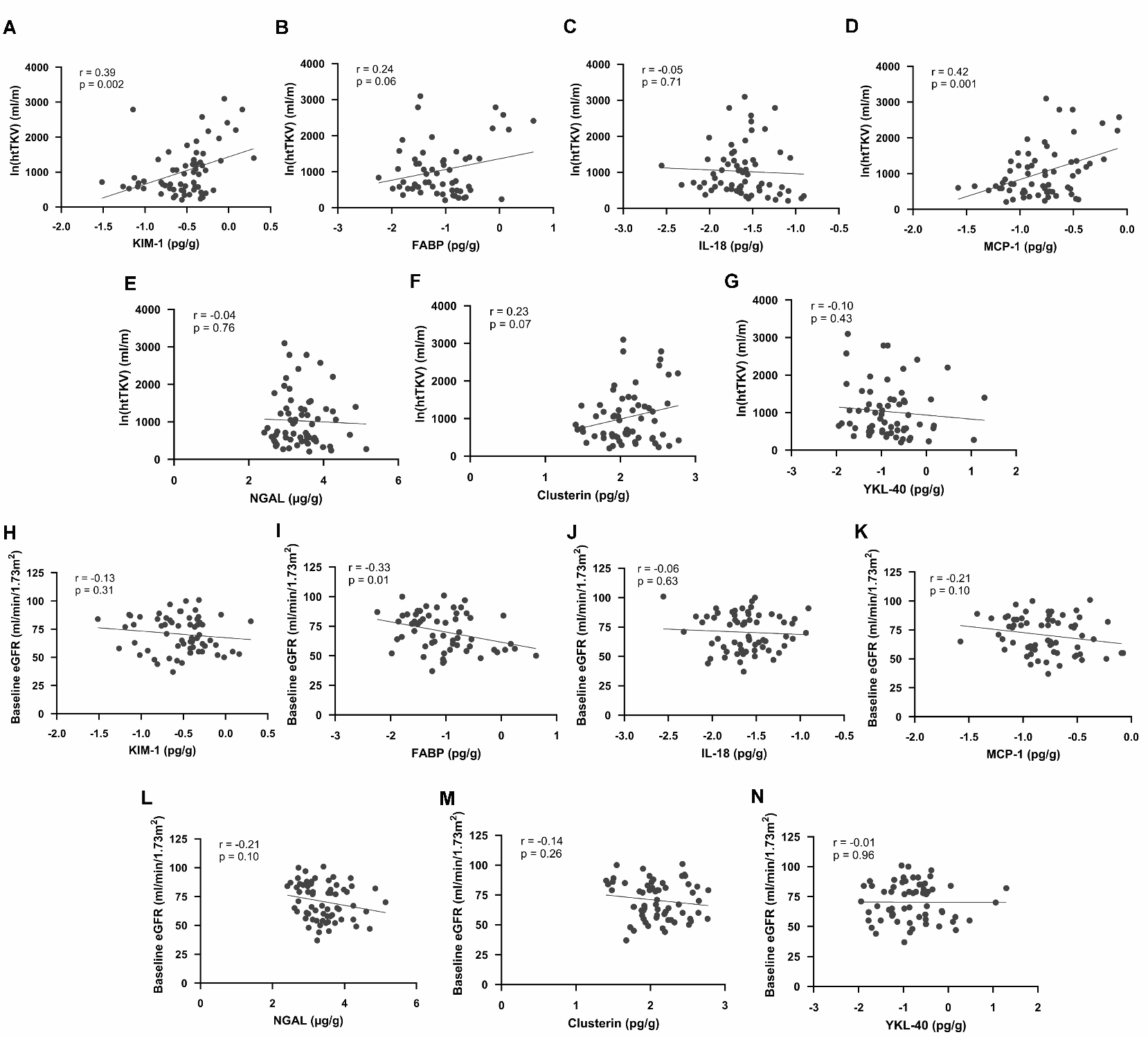


Bivariate correlation between urinary biomarkers and baseline (log-adjusted) height-adjusted total kidney volume (lnhTKV) (**A-G**) and baseline estimated glomerular filtration rate (eGFR) (**H-N**). Kidney injury molecule-1 (KIM-1; **Panel A** and **H**); fatty-acid binding protein 4 (FABP4; **Panel B** and **I**); interleukin-18 (IL-18; **Panel C** and **J**); monocyte chemoattractant protein-1 (MCP-1; **Panel D** and **K**); neutrophil gelatinase-associated lipocalin (NGAL; **Panel E** and **L**); clusterin (**Panel F** and **M**); human cartilage glycoprotein-40 (YKL-40; **Panel G** and **N**); all markers are normalized to urinary creatinine and log-transformed.
